# Use of androgens at different stages of life: reproductive period: Number 11 - December 2021

**DOI:** 10.1055/s-0041-1740610

**Published:** 2021-12-21

**Authors:** Andrea Prestes Nácul, Gabriela Pravatta Rezende, Daniela Angerame Yela Gomes, Técia Maranhão, Laura Olinda Bregieiro Fernandes Costa, Fernando Marcos dos Reis, Gustavo Arantes Rosa Maciel, Lia Cruz Vaz da Costa Damásio, Ana Carolina Japur de Sá Rosa e Silva, Vinicius Medina Lopes, Maria Cândida Baracat, Gustavo Mafaldo Soares, José Maria Soares, Cristina Laguna Benetti-Pinto

**Affiliations:** 1 Unidade de Reprodução Humana, Hospital Fúmina, Grupo Hospitalar Conceição, Porto Alegre,RS, Brazil; 2 Universidade Estadual de Campinas, Campinas, SP, Brazil; 3 Universidade Estadual de Campinas, Campinas, SP, Brazil; 4 Universidade Federal do Rio Grande do Norte, Natal, RN, Brazil; 5 Universidade de Pernambuco, Recife, PE, Brazil; 6 Universidade Federal de Minas Gerais, Belo Horizonte, MG, Brazil; 7 Faculdade de Medicina, Universidade de São Paulo, São Paulo, SP, Brazil; 8 Universidade Federal do Piauí, Teresina, PI, Brazil; 9 Faculdade de Medicina de Ribeirão Preto, Ribeirão Preto, SP, Brazil; 10 Universidade de Brasília, Brasília DF, Brazil; 11 Faculdade de Medicina, Universidade de São Paulo, São Paulo, SP, Brazil; 12 Universidade Federal do Rio Grande do Norte, Natal, RN, Brazil; 13 Faculdade de Medicina, Universidade de São Paulo, São Paulo, SP, Brazil; 14 Universidade Estadual de Campinas, Campinas, SP, Brazil

## Key points

- Although there are several situations that can potentially lead to lower androgen levels, until now is not defined a syndrome or biochemical criteria to diagnose androgen deficiency in women. Benefits of androgen therapy in these situations are controversial.
- Girls with delayed puberty manifesting primarily as a lack of development of secondary sex characters and primary amenorrhea may be deficient in sex hormone production, including androgen production deficiency.
- Due to surgical menopause and premature ovarian failure, androgen levels may be reduced compared to age-matched normal controls.
- Young women with hypopituitarism, anorexia nervosa, and adrenal insufficiency may have lower serum androgen levels.
- The use of androgens and anabolic steroids for aesthetic purposes has increased considerably due to issues related to the cult of the body and beauty. However, this is associated with undesirable and potentially irreversible aesthetic manifestations, in addition to an increase in morbidity.

## Recommendations

- There is no evidence for the use of androgen therapy in adolescent women, even in cases of hypogonadism due to ovarian (premature ovarian failure) or central causes.
- There is no evidence for routine androgen therapy in adult women with hypopituitarism, hypogonadotropic hypogonadism, adrenal insufficiency and anorexia nervosa.
- Androgen therapy, preferably by the transdermal route, may be considered for women with premature ovarian insufficiency (POI) or surgical menopause complaining of female sexual dysfunction (FSD), in addition to estrogen therapy.
- The use of androgens and anabolic steroids for aesthetic purposes is not recommended.
- The measurement of total testosterone or the calculation of the free androgen index can be used to control androgen replacement in women, but the available laboratory techniques do not present adequate sensitivity. Although the mass spectrometry technique has greater sensitivity, it is costly, which limits the use in clinical practice.

## Background

In the female sex, androgen is produced by the ovaries and adrenals and by peripheral conversion. Circulating testosterone levels play an important role in musculoskeletal and cardiovascular health and sexual function (SF). 1 Some authors have tried to define an androgen deficiency syndrome in women with symptoms such as low libido and global decrease in desire, fantasies and arousal, in addition to fatigue and decreased wellbeing. 2 However, several international entities, such as the Endocrine Society, the American College of Obstetricians and Gynecologists (ACOG), the American Society for Reproductive Medicine (ASRM), the European Society of Endocrinology (ESE) and the International Menopause Society (IMS), through their recommendations for clinical practice, took a stand against the clinical and laboratory diagnosis of androgen deficiency given the lack of uniformity in clinical criteria and the lack of standardization of serum testosterone levels in women according to age group. 3 Considering the difficulty of diagnosis, the treatment by androgen replacement in climacteric women has more widespread indication in the presence of FSD, although other situations can potentially lead to lower androgen levels in premenopausal women and raise doubts about the need for replacement. 3 4 These situations include, for example, the use of androgens to maintain bone mass and prevent osteoporosis, POI, hypopituitarism and adrenal insufficiency.

This document will be divided into two parts, in which we will review current evidence of indications and contraindications for the use of androgens in different situations in a woman's life – during the reproductive period (part I) and during the climacteric period (part II).

## In women, is there an indication of androgen dosage for the diagnosis of androgen deficiency?

The most commonly dosed androgen in women is testosterone. In premenopausal women, testosterone levels do not vary significantly with cycle phase and there is a modest increase during the peak of luteinizing hormone (LH) in ovulatory cycles. 5 This hormone circulates in the body in three ways: bound to sex hormone binding globulin – SHBG (66%), bound to albumin (33%) and in free form (1%). The free form and the albumin-bound form are considered the active forms of testosterone. 6 Several situations can increase SHBG levels, leading to a decrease in the free fraction of testosterone. In these cases, the measurement of free testosterone or the calculation of the free androgen index are more suitable for evaluating circulating testosterone in its biologically active form. 7

The most widely used tests for measuring testosterone such as immunometric assays and radioimmunoassay, had limitations, with low accuracy for detecting physiologically low levels in women. Currently, the liquid chromatography-mass spectrometry assay has greater sensitivity for testosterone measurement, so it is considered the gold standard test. 7 8 However, the higher cost and low availability of this method together with the need for highly specialized personnel limit its use. Testosterone measurement results should be carefully analyzed because serum levels do not reflect concentration in target tissues nor the individual variability in the sensitivity of peripheral receptors. 9 In addition, clinical effects also depend on conversion of testosterone by aromatase and by 5ɑ-reductase into estrone and dihydrotestosterone, respectively. 10 There is no indication for the dosage of dihydrotestosterone.

Although there is an association between serum androgen levels with sexual desire, its low level in women does not reliably predict clinical symptoms. Furthermore, testosterone levels are not independent predictors of SF in women. Thus, there is no biochemical diagnostic criterion to diagnose androgen deficiency in women. 3

## First part: use of androgens in the reproductive period

### Is there evidence for the use of androgens during adolescence?

In adolescence, the activation of the hypothalamus-pituitary-ovarian axis stimulates the production of ovarian hormones, especially estrogens, responsible for the thelarche, pubarche and menarche in girls. In parallel, this activation also increases circulating testosterone levels, which, during adolescence and reproductive years, are produced in an equivalent way by theca cells and the adrenal cortex at a concentration of approximately 300 mcg per day. 11 12 Serum androgen levels are higher during adolescence, with a progressive decrease after the age of 25 years. 13 In this phase of life, they play an important role in normal puberty and skeletal growth.

In adolescence, skeletal growth and bone mass gain are influenced by estrogens and androgens, with an important interaction between androgens, growth hormone (GH) and insulin-like growth factor (IGF-1). 14 15 Androgenic deficiencies can result in delayed puberty, manifested mainly by the absence of development of secondary sexual characteristics and primary amenorrhea, with hormone replacement being the first choice of treatment. The use of estrogenic hormone therapy (HT) in adolescents diagnosed with hypogonadism is validated for pubertal stimulation, cardiovascular protection, and prevention of osteoporosis. 14 15 16 17 However, the use of androgens in this period of life is only validated for male delayed puberty to promote growth and gain in muscle mass. There is not strong enough evidence to support its administration in adolescent women.

Although previous studies with different androgen therapy regimens in this age group in women with Turner syndrome and pan-hypopituitarism have shown some positive results such as bone and muscle mass gain and improved quality of life, they do not present enough evidence to recommend its use in female adolescents. 18 19 20

Therefore, there is no recommendation for the use of androgenic therapy in adolescent girls in any formulation or route of administration due to the lack of evidence to confirm its efficacy and safety in this age group.

### When is the use of androgens indicated during the reproductive period?

#### Premature ovarian insufficiency and surgical menopause

Premature ovarian insufficiency and surgical menopause are two situations in which there is a reduction in androgen levels compared to women with preserved gonadal function of the same age, as well as in comparison to women with natural menopause. 21 The addition of androgens in HT in women with POI does not seems to significantly improve quality of life, self-esteem or mood. The addition of testosterone to HT, although with limited evidence, does not seem to add benefits to bone mass either. On the other hand, it also does not seem to increase adverse events or effects such as skin changes, hirsutism, acne or frequency of waxing. 22 Premenopausal women undergoing bilateral oophorectomy usually present sexual dysfunctions related to low hormone levels. Evidence demonstrates an improvement in SF with the addition of testosterone to HT in these cases, preferably by transdermal route with minimal or mild adverse effects. However, the long-term effects are still unclear. 23 24 25 Although in Brazil, there are no products approved by regulatory agencies for the prescription of testosterone, the use of manipulated products is accepted, highlighting the difficulty of dose control. Testosterone 1% formulated in a high absorption gel (eg Pentravan) can be prescribed for transdermal use at a dose of 0.5 g of gel per day for three to six months. As a suggestion, the prescription of testosterone 5 mg per mL in a measuring bottle containing 30 mL of gel with a release of 1 mL per day is recommended. This dose can be individualized with a variation between 1 and 5 mg. Dose testosterone before starting treatment, after three to six weeks of use and for as long as the treatment lasts to avoid supraphysiological plasma levels and monitor the onset of clinical signs of hyperandrogenism, because the clinical response does not always correlate with plasma testosterone levels. If there is improvement, reinforce to the patient the lack of evidence regarding efficacy and safety in use for more than 24 months. 23

#### Hypopituitarism

Hypopituitarism often includes hypogonadotropic hypogonadism and centrally-caused adrenal insufficiency, with a deficient production of androgens by the two major sources – ovaries and adrenals. A study that evaluated the use of 300 mcg testosterone patches for one year in 51 women with hypopituitarism showed an increase in bone mass in the hip and radius and in muscle mass, as well as improvement in mood and SF in this group of women. 24 If androgen therapy is considered for women with hypopituitarism, follow-up should be done in the same way as for women with FSD, although the long-term safety is unknown and there is no formal recommendation for this replacement. 3 25

#### Adrenal insufficiency

Women with adrenal insufficiency have lower levels of dehydroepiandrosterone (DHEA) and its sulfated form (SDHEA). Many women with primary and secondary adrenal insufficiency present deterioration in their general health status despite glucocorticoid and mineralocorticoid replacement. 26 Although some studies demonstrate that DHEA replacement improves depression and anxiety scores, feelings of wellbeing, libido and SF, a systematic review and meta-analysis showed only modest improvement in quality of life and depression scores. Dehydroepiandrosterone is a dietary supplement without regulatory control and robust evidence to indicate its routine use in adrenal insufficiency. 27 In practice, androgen replacement with DHEA is sometimes used for women with symptoms of androgen deficiency and low plasma levels of DHEA, with starting doses between 25 and 50 mg daily for a period of three to six months and dose adjustments according to circulating DHEA levels and clinical symptoms. In the absence of a satisfactory therapeutic result or in the presence of adverse effects, therapy should be suspended. 28

#### Anorexia nervosa

Women with anorexia nervosa have lower total and free testosterone levels than women without this condition. 29 Free testosterone levels have a positive correlation with body mass index (BMI) and spinal bone mineral density and a negative correlation with depression levels in these women. 30 Although studies have shown higher bone formation markers with the use of testosterone patches, this increase did not reflect in an increase in bone mass. 29 30 Although there may be an increase of lean mass, the use of testosterone can even lead to a decrease in BMI. 31 Testosterone replacement also did not show positive effects on depression and anxiety indices. 31 Thus, testosterone replacement should not be recommended for the prevention of bone mass loss, weight gain and emotional disorders associated with anorexia nervosa.

### What can we say about the use of anabolic androgens for aesthetic/recreational purposes?

The current exaggerated cult of the body and aesthetic beauty has made many people seek quick results through substance abuse with the promise of better body image and physical performance. Among these substances are androgenic anabolic steroids (AAS), 32 including testosterone and its synthetic derivatives. Androgenic anabolic steroids are typically given in supraphysiological doses for periods called cycles, or used continuously with constant or varying doses. These substances are efficient in promoting increase in muscle size and strength, in the search for an idealized body image, as a result of cultural stimuli or, for some, as a result of body dysmorphic disorders. 33

Empirical evidence suggested that AAS were used primarily by top-level competitive athletes and especially weightlifters, bodybuilders, and track athletes. Currently, AAS are widely used not only by athletes involved in recreational and minor league sports, but also by non-athletes. 34

In Brazil, gestrinone, previously used for the treatment of endometriosis and discontinued because of its androgenic side effects, is now being marketed again in the form of an implant and called “beauty chip”, given a possible aesthetic effect )#x0028;reduction of body fat and promotion of lean mass gain). There are no studies evaluating gestrinone for improved libido or lean mass gain, as well as its long-term effects, such as possible carcinogenic risk or secondary infertility. This product is not approved for use by the National Health Surveillance Agency (Anvisa), and the Federal Council of Medicine only recommends the use of hormonal implants for medical purposes, given the lack of any evidence of the promised aesthetic benefits.

Users may associate different AAS, as well as with other potentially anabolic products such as GH and insulin for better results, although with an increase in androgenic and metabolic effects. 35

In women, the most frequent alterations attributed to AAS abuse are menstrual irregularities (late menarche, oligomenorrhea, secondary amenorrhea), dysmenorrhea, anovulation, acne, alopecia, clitoris hypertrophy, libido alterations, breast tissue atrophy and uterine atrophy, many of which are permanent. 36 In adolescents, it can cause early skeletal maturation with closure of the bony epiphyses, short stature and accelerated puberty, leading to dysmorphic growth. The use of AAS has been associated with a number of medical and psychological side effects, including mental health and cognitive function disorders, metabolic and endocrine disorders, and cardiovascular pathology. 37 38

Therefore, we do not recommend the use of AAS for aesthetic purposes. This use is illegal, can cause addiction, and numerous harmful side effects.

### Is there an indication for the use of androgens in users of combined oral contraceptives (COCs)?

The effects of COCs on SF remain controversial. In order to improve SF, some authors have proposed the concomitant use of androgens, especially DHEA. The exchange of ethinylestradiol for 17β-estradiol is also suggested, with a lower elevation of SHBG and a higher index of free androgens. However, there is lack of evidence on the impact of COCs on SF, and on the fact that this dysfunction is caused by a reduction in androgenic activity. 39

## Final considerations

There is limited evidence on the use of androgens in women during the age compatible with the reproductive period, indicating their use in situations of bilateral oophorectomy and premature ovarian insufficiency. There is a lack of evidence to support other indications. The use of androgens for aesthetic purposes should not be recommended.

National Specialty Commission on Gynecology Endocrinology of the Brazilian Federation of Gynecology and Obstetrics Associations (FEBRASGO)

President:

Cristina Laguna Benetti Pinto

Vice-President:

Ana Carolina Japur de Sá Rosa e Silva

Secretary:

José Maria Soares Júnior

Members:

Andrea Prestes Nácul

Daniela Angerame Yela

Fernando Marcos dos Reis

Gabriela Pravatta Rezende

Gustavo Arantes Rosa Maciel

Gustavo Mafaldo Soares

Laura Olinda Rezende Bregieiro Costa

Lia Cruz Vaz da Costa Damásio

Maria Candida Pinheiro Baracat Rezende

Sebastião Freitas de Medeiros

Tecia Maria de Oliveira Maranhão

Vinicius Medina Lopes
